# Time course of neuropsychological functioning after aneurysmal subarachnoid hemorrhage

**DOI:** 10.1186/cc12282

**Published:** 2013-03-19

**Authors:** B Zarino, G Bertani, V Conte, S Magnoni, A Di Cristofori, N Stocchetti

**Affiliations:** 1 Fondazione IRCCS Ca' Granda-Ospedale Maggiore Policlinico, Milan, Italy; 2 Fondazione IRCCS Ca' Granda-Ospedale Maggiore Policlinico, Milan University, Milan, Italy

## Introduction

Early and delayed cognitive dysfunctions are an understudied issue after aneurysmal subarachnoid hemorrhage (aSAH), irrespective of neurological outcome. The aim of this study was to describe early and delayed changes of cognitive functions, activities of everyday life, and quality of life in aSAH patients.

## Methods

Consecutive aSAH patients admitted to our ICU between November 2011 and September 2012 were prospectively studied. Patients underwent neuropsychological evaluation at early (<3 days, 10 days) and delayed time points (1 month, 3 months). Patients were tested for language, verbal fluency, short-term and long-term memory, attention, executive functions, praxis, and neglect. Impairments in activities of everyday life were assessed using the Activities of Daily Living scale and the Instrumental Activities of Daily Living scale. The SF-36 was used to assess the quality of life at 3 months. Since complications of aneurysm treatment in addition to aSAH severity may significantly affect cognitive status, patients were evaluated according to the World Federation of Neurological Surgeons score after treatment (WFNSpt).

## Results

All WFNSpt 1 to 2 patients completed neuropsychological tests at each time point. WFNSpt 3 and WFNS 4 patients were testable in 80% and 50% of the cases respectively at early time points. WFNS 5 patients were not testable at any time point. In all testable patients, cognitive functions were severely impaired at early time points. At 3 months in WFNS 1 to 3 a good recovery of language deficits while only a partial recovery of attention, memory and executive functions were observed; at the same time point 70% of WFNS 4 patients became testable, but they had a worse recovery of all cognitive functions. At 1 month after SAH less than 30% of patients return to work, at 3 months approximately 50%. Despite a good recovery of everyday life activities at 3 months, for all patients quality of life was lower than a normal population.

## Conclusion

Cognitive dysfunction has different time courses after aSAH: significant deficits in different cognitive domains, worse quality of life and difficulties in return to work persist in more than 50% of patients at 3 months following SAH.

